# Intercellular Adhesion Molecule and Endogenous NOS Inhibitor: Asymmetric Dimethylarginine in Pregnant Women with Gestational Diabetes Mellitus

**DOI:** 10.1155/2016/1342643

**Published:** 2016-02-11

**Authors:** Elżbieta Poniedziałek-Czajkowska, Radzisław Mierzyński, Dariusz Szymula, Bożena Leszczyńska-Gorzelak, Jan Oleszczuk

**Affiliations:** Chair and Department of Obstetrics and Perinatology, Medical University of Lublin, Jaczewskiego 8, 20-954 Lublin, Poland

## Abstract

*Objective*. The aim of the study was to evaluate the concentrations of soluble intercellular adhesion molecule-1 (s-ICAM-1) and endogenous NOS inhibitor, asymmetric dimethylarginine (ADMA), as markers of endothelium dysfunction in patients with gestational diabetes mellitus (GDM).* Patients and Methods*. The levels of s-ICAM-1 and ADMA were analysed in the group of 56 patients with GDM and compared to 25 healthy pregnant women. The concentrations of s-ICAM-1 and ADMA were measured in serum using ELISA tests.* Results*. The groups did not differ by baseline descriptors: age (30.75 ± 6.32 versus 28.50 ± 4.95 years, NS) and gestational age (28.96 ± 2.85 versus 29.12 ± 2.96 hbd, NS). The patients with GDM were more obese (BMI 27.93 ± 7.02 versus 22.34 ± 4.21 kg/m^2^, *p* = 0.032) and had higher concentration of C-reactive protein (6.46 ± 6.03 versus 3.18 ± 3.83 mg/L, *p* = 0.029). In the GDM group the level of ADMA was lower (0.38 ± 0.17 versus 0.60 ± 0.28 *μ*mol/L, *p* = 0.001) and the level of s-ICAM-1 was significantly higher (289.95 ± 118.12 versus 232.56 ± 43.31 ng/mL, *p* = 0.036) compared to controls.* Conclusions*. The pregnant women with GDM are characterized by higher concentration of s-ICAM-1 that reflects the activation and dysfunction of the endothelial cells. The decreased ADMA level in GDM patients seems to be preventive in the limitation of NO synthesis caused by the impaired insulin action and the endothelial dysfunction.

## 1. Introduction

The physiological pregnancy induces insulin resistance (IR) which appears to result from a combination of increased maternal adiposity and the insulin-desensitizing effects of placental hormones [1]. It is suggested that there are some possible etiological mechanisms responsible for GDM development. Women with GDM are often obese so processes linking the obesity to insulin resistance and/or promoting obesity are likely to play an important role. There is a lot of evidence that endothelial dysfunction may be responsible for IR. It is defined as the partial or complete loss of balance between vasoconstrictors and vasodilators, growth promoting and inhibiting factors, proatherogenic and antiatherogenic factors, and procoagulant and anticoagulant factors [2]. Most frequently the term “endothelial dysfunction” has been used to refer to the impaired endothelium-dependent relaxation caused by loss of nitric oxide (NO) bioactivity in the vessel wall [3]. Nitric oxide not only produces vasodilation, but also participates in various processes that are beneficial to the vasculature, such as reduction of the vascular smooth muscle cell migration and growth, platelets aggregation and thrombosis, and monocytes and macrophages adhesion and inflammation. One of the methods of assessment of endothelial function is to measure the levels of endothelial activation markers, such as soluble vascular cell adhesion molecule (sVCAM), soluble intercellular adhesion molecule (sICAM), endothelin 1 (ET-1), E-selectin, and others, such as markers of the coagulation/fibrinolysis and low-grade inflammation. sICAM-1 is a member of the immunoglobulin superfamily that mediates its functional activity through binding to leukocyte 2-integrins. The sICAM-1 molecule is functionally involved in the regulation of adhesion of leukocytes to the endothelium as well as leukocytes migration [4]. Both the impairment of glucose tolerance and type 2 diabetes are characterized by higher plasma levels of sICAM [5]. The bioavailability of nitric oxide is a key factor for the development of endothelial dysfunction and, furthermore, it seems to play an important role in the development of insulin resistance. Insulin itself, in addition to its metabolic actions, has a direct influence on the endothelium and smooth muscle cells and increases the skeletal muscle blood flow in healthy, insulin-sensitive people. Endothelium-derived NO synthase (eNOS) regulates blood flow to insulin-sensitive tissues (i.e., skeletal muscle, liver, and adipose tissue), and its activity is impaired in insulin-resistant individuals. The inhibition of eNOS disturbs microvascular recruitment and blunts muscle glucose uptake in response to insulin [6]. There is growing evidence that the increased level of eNOS inhibitor, asymmetric dimethylarginine (ADMA), which is a posttranslationally modified form of arginine, contributes to the endothelial dysfunction and diminishes endothelium-related vasodilatation [7]. The raised ADMA plasma concentration may contribute to the vascular defect observed in the IR and is associated with abnormalities closely related to this syndrome such as hypertriglyceridemia, hyperglycaemia, and hypertension. The ADMA level is positively correlated with the impairment of insulin-mediated glucose disposal in nondiabetic, normotensive individuals [8]. During normal pregnancy ADMA diminishes, but it increases in women with preeclampsia [9]. A clear correlation with endothelial dysfunction has been reported in women with high levels of ADMA at the beginning of pregnancy [10].

Both ADMA and s-ICAM-1 are thought to be the markers of endothelial dysfunction. Extensive literature on their role in various pathologies in humans is available but so far there has been little evidence related to their significance in GDM. Thus, the aim of this study was to analyse the serum levels of ADMA and s-ICAM-1 as the markers of endothelium dysfunction in patients with gestational diabetes.

## 2. Patients and Methods

Fifty-six women with GDM and 25 healthy pregnant women were included in the study, which was performed in the Department of Obstetrics and Perinatology at Medical University of Lublin. All of them signed informed consent to participate in the study, which had previously been approved by the Bioethical Review Board of the Medical University in Lublin. The inclusion criteria were as follows: gestational age between 24th and 34th week as determined on ultrasound examination before 20th week of gestation, first prenatal visit before 8th week, singleton pregnancy, and gestational diabetes mellitus first diagnosed in the current pregnancy before completion of 28 weeks of gestation. The exclusion criteria were as follows: prepregnancy diabetes mellitus, any form of hypertension, prepregnancy and gestational hypertension, preeclampsia, chronic renal disease, intrauterine growth retardation, liver diseases, inflammation, and infectious diseases; patients with positive urine or vaginal culture were also excluded from the study as well as cigarette smokers. The oral glucose tolerance test (OGTT) with 75 g of glucose in compliance with WHO standards was performed in all women participating in the study and in the control group between 24th and 28th week of gestation. The gestational diabetes mellitus was diagnosed on the basis of the following WHO criteria: fasting ≥92 mg/dL (5.1 mmol/L), at 1st hour ≥180 mg/dL (10.0 mmol/L) and at 2nd hour ≥153 mg/dL (8.5 mmol/L) [11].

The glucose concentration was measured in venous blood plasma with the use of glucose oxidase method (Cormay, Poland). The ADMA, s-ICAM-1, C-reactive protein levels were assessed. The blood samples for research tests were taken from patients who were fasting together with the samples for routinely performed laboratory tests. The samples were allowed to clot for at least 30 minutes before centrifugation at 1000 G, which was continued for 20 minutes. Serum has been removed and then frozen at −70°C. The s-ICAM-1 concentration was measured by means of the sandwich enzyme immunoassay technique (Human sICAM-1 Immunoassay, R&D Systems Inc. Minneapolis, USA) as well as the ADMA level (ADMA direct ELISA Kit, Immundiagnostik AG, Bensheim, Germany). CRP concentration was measured with high-sensitivity CRP assay (CRP HS ELISA, DRG International, Inc., USA). Body mass index (BMI) calculation was based on the weight measurement at the first prenatal visit before 8th week of gestation.

The study group was compared to the control one with respect of maternal age, gestational age at the entry to the study, parity, BMI, CRP levels, and glucose levels at each hour of OGTT as well as s-ICAM-1 and ADMA concentrations. Correlations between ADMA and s-ICAM-1 and BMI, CRP, and glucose levels at each hour of OGTT were analysed.

The Statistica 5.5A for Windows (StatSoft, Poland) was used for data analysis. The elements of descriptive statistics were used. Data was presented as mean ± standard deviation. The Shapiro-Wilk test for normal distribution of data and one-tailed Student's* t*-test, or (in unequal variance) the Cochran-Cox test (absence of normal distribution and nonparametric data), and the Mann-Whitney *U* test were performed. The Spearman rank test was employed for searching correlations between variables. The statistical significance was defined as *p* < 0.05.

## 3. Results

The studied groups did not differ with regard to the baseline descriptors: age, gestational age when included to the study, and parity (Table 1). Patients with GDM were more obese than the controls (BMI 27.93 ± 7.02 versus 22.34 ± 4.21 kg/m^2^, *p* = 0.032) and had higher concentration of C-reactive protein (6.46 ± 6.03 versus 3.18 ± 3.83 mg/L, *p* = 0.029) (Table 1). The glucose levels during OGTT fasting, at 1st and 2nd hour of test, were significantly higher in GDM group (0′: 93.35 ± 18.28 versus 78.88 ± 6.62 mg/dL, *p* = 0.001; 1′: 181.50 ± 21.29 versus 107.29 ± 22.60 mg/dL, *p* < 0.000001; 2′: 165.39 ± 29.42 versus 83.89 ± 28.13 mg/dL, *p* < 0.000001) (Table 2). The level of ADMA was lower in GDM group in comparison with the controls (0.38 ± 0.17 versus 0.60 ± 0.28 *μ*mol/L, *p* = 0.001), but the level of s-ICAM-1 was significantly higher in patients with GDM (289.95 ± 118.12 versus 232.56 ± 43.31 ng/mL, *p* = 0.036) (Table 2).

No correlations were found between ADMA and s-ICAM-1 levels and BMI, CRP, and glucose levels both fasting and at the 1st hour of OGTT in the GDM patients as well as in the control ones. A marked, but statistically insignificant, correlation of ADMA level and the glucose level at the 2nd hour of OGTT was observed in patient with GDM (Table 3).

## 4. Discussion

The insulin resistance increases during pregnancy and in the third trimester, and it is comparable to this observed in type 2 diabetes. It is partly related to the growth of adiposity, but its sudden drop right after delivery is suggestive of an important role of placental hormones acting as antagonists of insulin. Development of GDM only in some pregnant women indicates the role of other factors like prepregnancy insulin resistance observed in obesity and/or endothelium dysfunction. Being overweight before conception adds an already existing insulin resistance (both hepatic and peripheral) to the normal changes of the glucose tolerance present in late gestation. There is a 40% reduction in peripheral insulin sensitivity in obese pregnant women and insulin response to an intravenous glucose load is reduced in obese women in comparison with lean pregnant ones [12]. Obesity was found to be associated with the elevated s-ICAM-1 levels [13]. We observed that patients with GDM were overweight and had significantly higher concentration of s-ICAM-1 compared to healthy controls. They also had significantly higher levels of C-reactive protein. No correlation was noted between the s-ICAM-1 levels and BMI, CRP levels, or glucose levels in OGTT either in GDM or control group. These results suggest activation and dysfunction of endothelial cells as well as the inflammation in course of GDM. In vitro, insulin was shown to decrease ICAM-1 expression [14]. In type 2 diabetic patients, s-ICAM-1 levels were found to be increased [15]. In the large Nurses' Health Study that included 121,700 subjects, s-ICAM-1 level was predictive for the incidence of diabetes [16]. Therefore, it might be postulated that not only obesity, inflammation, or glucose metabolism impairment's degree could result in endothelium activation in GDM. There is little evidence about s-ICAM role in GDM; most studies reveal the significance of s-ICAM-1 as predictor of type 2 diabetes in women with previous GDM. It was shown that, despite resolving of previous metabolic anomalies, higher levels of ICAM and VICAM were observed after delivery in women with previous GDM compared to healthy ones. This appears to suggest persistent endothelium dysfunction which coexists with an increased future cardiovascular risk [17]. ICAM-1 and VICAM-1 have been reported to predict the development of type 2 diabetes in women irrespective of other known risk factors including abdominal obesity or CRP levels as a marker of inflammation [16]. There is a lot of evidence emphasizing the predictive role of ICAM i VICAM in early identification of patients in whom preeclampsia develops [4, 18].

In NO synthesis, insulin and ADMA have opposite roles. There is a growing evidence that increased level of ADMA contributes to the endothelial dysfunction and diminishes endothelium-related vasodilatation. ADMA inhibits eNOS and diminishes NO synthesis, whereas insulin is known to have a direct vasodilatory effect mediated through stimulation of NO production in the endothelial cells [19]. In the insulin-resistant state, the ability of insulin to stimulate NO production in the endothelium is diminished. It may be assumed that the excess of ADMA can suppress insulin-related vasodilatation and promotes insulin resistance development [20]. In this study it was shown that patients with GDM are characterized by significantly lower levels of ADMA when compared to healthy pregnant women. No correlations were found between ADMA level and BMI, CRP level, and glucose levels fasting at 1st hour of OGTT. A marked but statistically insignificant correlation of ADMA level and the glucose level at 2nd hour of OGTT was observed. There is a lot of evidence that raised ADMA concentrations may contribute to the vascular pathology observed in the insulin resistance [8, 21]. There is published data indicating that ADMA concentrations are significantly higher in GDM patients in comparison with the healthy controls [22, 23]. Therefore, in this context the results of this study, which showed lower ADMA levels in GDM group, appear to be surprising. However, it was also suggested that chronic hyperglycaemia might downregulate mechanisms implicated in ADMA production or stimulate its metabolism [24]. According to the study of Anderssohn et al. the relationship between ADMA and diabetes seems to be ambiguous and partly related to type and stage of DM [25].

In the course of physiological pregnancy ADMA levels decline even with an elevated concentration of total cholesterol, LDL and HDL cholesterol, and triglycerides. Pathological stimuli such as hyperglycaemia, inflammatory cytokines, hyperhomocysteinaemia, infectious agents, and oxidized low-density lipoprotein (LDL) may reduce activity of degrading ADMA enzyme, dimethylarginine dimethylaminohydrolase (DDAH), and allow ADMA to accumulate [8, 26]. Lower ADMA levels during normal pregnancy may be explained with increase in DDAH activity [27] or they may be reflective of the physiological adaptation to pregnancy, irrespective of the disturbances of glucose tolerance [23]. Our observations are similar to the results of study presented by Takaya et al. who found that the level of ADMA was significantly lower among children with diabetes and obesity compared to control subjects and that ADMA concentrations were inversely correlated with serum glucose level. It has been suggested that limited ADMA synthesis could be protective against the endothelial cell dysfunction caused by hyperglycaemia which is partly related to the limited NO synthesis [28].

It has been postulated that hyperglycaemia could decrease ADMA levels through the restricted ADMA synthesis or through enhancing its metabolism by the enzyme DDAH [29]. Additionally, it is widely known that the prolonged impact of high glucose levels on endothelial cells increases NO synthase expression and superoxide anion generation [30]. Thus, some other factors responsible for ADMA lowering in pregnancy should be taken into consideration. It has been proven that estrogens that have antioxidant properties may increase the activity of DDAH what is accompanied by a reduced release of ADMA. The estrogens synthesis increases as pregnancy progresses, but some other factors should be considered as causative since ADMA concentration in GDM is even lower than in healthy pregnancy [31]. It may be presumed that lowering ADMA levels in GDM patients might result from excellent glucose control after GDM diagnosis. Das et al. observed that the strict control of hyperglycaemia decreases the serum ADMA levels [32]. Consequently, a hypothesis could be formulated that decreased ADMA levels in GDM could be preventive in the limitation of NO synthesis due to impaired insulin action.

## 5. Conclusions

Pregnant women with GDM are characterized by higher concentration of s-ICAM-1 which reflects the activation and dysfunction of the endothelial cells. The decreased ADMA level in GDM patients seems to be preventive in the limitation of NO synthesis caused by the impaired insulin action and the endothelial dysfunction.

## Figures and Tables

**Table 1:** Characteristics of group with GDM and control.

	GDM group	Control group	*p*
	*N* = 56	*N* = 25	
Age (years)	30.75 ± 6.32	28.50 ± 4.95	NS
Gestational age (hbd)	28.69 ± 2.85	29.13 ± 2.96	NS
Parity	2.14 ± 1.22	1.88 ± 1.09	NS
BMI (kg/m^2^)	27.93 ± 7.02	22.34 ± 4.21	*p* = 0.032
CRP (mg/L)	6.46 ± 6.03	3.18 ± 3.83	*p* = 0.029

GDM: gestational diabetes mellitus.

BMI: body mass index.

CRP: C-reactive protein.

*p*: statistical significance.

NS: statistically not significant.

**Table 2:** ADMA, s-ICAM-1, and glucose levels in GDM and control group.

	GDM group	Control group	*p*
	*N* = 56	*N* = 25	
ADMA (*μ*mol/L)	0.38 ± 0.17	0.60 ± 0.28	*p* = 0.001
s-ICAM-1 (ng/mL)	289.95 ± 118.12	232.56 ± 43.31	*p* = 0.036
Glucose (mg/dL)			
0′	93.35 ± 18.28	78.88 ± 6.62	*p* = 0.001
1′	181.50 ± 21.29	107.29 ± 22.60	*p* < 0.000001
2′	165.39 ± 29.42	83.89 ± 28.13	*p* < 0.000001

GDM: gestational diabetes mellitus.

ADMA: asymmetric dimethylarginine.

s-ICAM-1: soluble intercellular adhesion molecule-1.

*p*: statistical significance.

**Table 3:** Correlation between ADMA and s-ICAM-1 levels and BMI and CRP levels and glucose levels in OGTT in patients with GDM and control group.

	GDM				Control			
	ADMA		s-ICAM-1		ADMA		s-ICAM-1	
	*R*	*p*	*R*	*p*	*R*	*p*	*R*	*p*
BMI	−0.067	NS	0.309	NS	0.160	NS	−0.397	NS
CRP	0.058	NS	0.261	NS	−0.290	NS	0.094	NS
Glucose								
0′	−0.156	NS	−0.090	NS	−0.148	NS	−0.071	NS
1′	−0.167	NS	−0.050	NS	−0.205	NS	0.240	NS
2′	0.326	*p* = 0.05	0.285	NS	−0.368	NS	0.011	NS
ADMA			−0.044	NS			0.122	NS

GDM: gestational diabetes mellitus.

BMI: body mass index.

CRP: C-reactive protein.

ADMA: asymmetric dimethylarginine.

s-ICAM-1: soluble intercellular adhesion molecule-1.

*R*: Spearman correlation's coefficient.

*p*: statistical significance.

NS: statistically not significant.

## References

[1] Buchanan T. A., Xiang A. H. (2005). Gestational diabetes mellitus. *The Journal of Clinical Investigation*.

[2] Quyyumi A. A. (1998). Endothelial function in health and disease: new insights into the genesis of cardiovascular disease. *American Journal of Medicine*.

[3] Cai H., Harrison D. G. (2000). Endothelial dysfunction in cardiovascular diseases: the role of oxidant stress. *Circulation Research*.

[4] Kim S.-Y., Ryu H.-M., Yang J. H. (2004). Maternal serum levels of VCAM-1, ICAM-1 and E-selectin in preeclampsia. *Journal of Korean Medical Science*.

[5] Matsumoto K., Sera Y., Nakamura H., Ueki Y., Miyake S. (2002). Serum concentrations of soluble adhesion molecules are related to degree of hyperglycemia and insulin resistance in patients with type 2 diabetes mellitus. *Diabetes Research and Clinical Practice*.

[6] Vincent M. A., Barrett E. J., Lindner J. R., Clark M. G., Rattigan S. (2003). Inhibiting NOS blocks microvascular recruitment and blunts muscle glucose uptake in response to insulin. *The American Journal of Physiology—Endocrinology and Metabolism*.

[7] Kielstein J. T., Impraim B., Simmel S. (2004). Cardiovascular effects of systemic nitric oxide synthase inhibition with asymmetrical dimethylarginine in humans. *Circulation*.

[8] Lin K. Y., Ito A., Asagami T. (2002). Impaired nitric oxide synthase pathway in diabetes mellitus: role of asymmetric dimethylarginine and dimethylarginine dimethylaminohydrolase. *Circulation*.

[9] López-Alarcón M., Montalvo-Velarde I., Vital-Reyes V. S., Hinojosa-Cruz J. C., Leaños-Miranda A., Martínez-Basila A. (2015). Serial determinations of asymmetric dimethylarginine and homocysteine during pregnancy to predict pre-eclampsia: a longitudinal study. *BJOG: An International Journal of Obstetrics & Gynaecology*.

[10] Alpoim P. N., Godoi L. C., Freitas L. G., Gomes K. B., Dusse L. M. (2013). Assessment of L-arginine asymmetric 1 dimethyl (ADMA) in early-onset and late-onset (severe) preeclampsia. *Nitric Oxide*.

[11] WHO (2013). Diagnostic criteria and classification of hyperglycaemia first detected in pregnancy. *WHO/NMH/MND*.

[12] Catalano P. M., Huston L., Amini S. B., Kalhan S. C. (1999). Longitudinal changes in glucose metabolism during pregnancy in obese women with normal glucose tolerance and gestational diabetes mellitus. *American Journal of Obstetrics and Gynecology*.

[13] Ito H., Ohshima A., Inoue M. (2002). Weight reduction decreases soluble cellular adhesion molecules in obese women. *Clinical and Experimental Pharmacology and Physiology*.

[14] Hamaguchi T., Namba M. (2003). Insulin therapy in diabetic patient with hypertension. *Nippon Rinsho*.

[15] Takeuchi N., Kawamura T., Kanai A. (2002). The effect of cigarette smoking on soluble adhesion molecules in middle-aged patients with Type 2 diabetes mellitus. *Diabetic Medicine*.

[16] Meigs J. B., Hu F. B., Rifai N., Manson J. E. (2004). Biomarkers of endothelial dysfunction and risk of type 2 diabetes mellitus. *The Journal of the American Medical Association*.

[17] Bo S., Valpreda S., Menato G. (2007). Should we consider gestational diabetes a vascular risk factor?. *Atherosclerosis*.

[18] Abe E., Matsubara K., Ochi H., Ito M., Oka K., Kameda K. (2003). Elevated levels of adhesion molecules derived from leukocytes and endothelial cells in patients with pregnancy-induced hypertension. *Hypertension in Pregnancy*.

[19] Kuboki K., Jiang Z. Y., Takahara N. (2000). Regulation of endothelial constitutive nitric oxide synthase gene expression in endothelial cells and in vivo: a specific vascular action of insulin. *Circulation*.

[20] Montagnani M., Quon M. J. (2000). Insulin action in vascular endothelium: potential mechanisms linking insulin resistance with hypertension. *Diabetes, Obesity and Metabolism*.

[21] Matsuoka H., Itoh S., Kimoto M. (1997). Asymmetrical dimethylarginine, an endogenous nitric oxide synthase inhibitor, in experimental hypertension. *Hypertension*.

[22] Akturk M., Altinova A., Mert I. (2010). Asymmetric dimethylarginine concentrations are elevated in women with gestational diabetes. *Endocrine*.

[23] Telejko B., Zonenberg A., Kuzmicki M. (2009). Circulating asymmetric dimethylarginine, endothelin-1 and cell adhesion molecules in women with gestational diabetes. *Acta Diabetologica*.

[24] Marcovecchio M. L., Widmer B., Turner C., Dunger D. B., Dalton R. N. (2011). Asymmetric dimethylarginine in young people with Type 1 diabetes: a paradoxical association with HbA_1c_. *Diabetic Medicine*.

[25] Anderssohn M., Schwedhelm E., Lüneburg N., Vasan R. S., Böger R. H. (2010). Asymmetric dimethylarginine as a mediator of vascular dysfunction and a marker of cardiovascular disease and mortality: an intriguing interaction with diabetes mellitus. *Diabetes and Vascular Disease Research*.

[26] Leiper J., Murray-Rust J., McDonald N., Vallance P. (2002). S-nitrosylation of dimethylarginine dimethylaminohydrolase regulates enzyme activity: further interactions between nitric oxide synthase and dimethylarginine dimethylaminohydrolase. *Proceedings of the National Academy of Sciences of the United States of America*.

[27] Murray-Rust J., Leiper J., McAlister M. (2001). Structural insights into the hydrolysis of cellular nitric oxide synthase inhibitors by dimethylarginine dimethylaminohydrolase. *Nature Structural Biology*.

[28] Takaya J., Tanabe Y., Kuroyanagi Y., Kaneko K. (2015). Asymmetric dimethylarginine is negatively correlated with hyperglycemia in children. *Endocrine Journal*.

[29] Ellger B., Richir M. C., Van Leeuwen P. A. M. (2008). Glycemic control modulates arginine and asymmetrical-dimethylarginine levels during critical illness by preserving dimethylarginine-dimethylaminohydrolase activity. *Endocrinology*.

[30] Cosentino F., Hishikawa K., Katusic Z. S., Lüscher T. F. (1997). High glucose increases nitric oxide synthase expression and superoxide anion generation in human aortic endothelial cells. *Circulation*.

[31] Dai Z., Zhu H.-Q., Jiang D.-J., Jiang J.-L., Deng H.-W., Li Y.-J. (2004). 17*β*-Estradiol preserves endothelial function by reduction of the endogenous nitric oxide synthase inhibitor level. *International Journal of Cardiology*.

[32] Das U. N., Repossi G., Dain A., Eynard A. R. (2011). L-arginine, NO and asymmetrical dimethylarginine in hypertension and type 2 diabetes. *Frontiers in Bioscience*.

